# Association Studies of *ERCC1* Polymorphisms with Lung Cancer Susceptibility: A Systematic Review and Meta-Analysis

**DOI:** 10.1371/journal.pone.0097616

**Published:** 2014-05-19

**Authors:** Jinhong Zhu, Rui-Xi Hua, Jing Jiang, Li-Qin Zhao, Xiuwei Sun, Jinwei Luan, Yaoguo Lang, Yanqi Sun, Kun Shang, Shiyun Peng, Jianqun Ma

**Affiliations:** 1 Molecular Epidemiology Laboratory, Harbin Medical University Cancer Hospital, Harbin, Heilongjiang, China; 2 Department of Oncology, The First Affiliated Hospital of Sun Yat-Sen University, Guangzhou, Guangdong, China; 3 Department of Oncology, Harbin Medical University Cancer Hospital, Harbin, Heilongjiang, China; 4 Department of Hematology, The First Affiliated Hospital, University of South China, Hengyang, Hunan, China; 5 Department of Radiology, Harbin Medical University Cancer Hospital, Harbin, Heilongjiang, China; 6 Department of Thoracic Surgery, Harbin Medical University Cancer Hospital, Harbin, Heilongjiang, China; 7 Department of Nuclear Medicine, Xuanwu Hospital, Capital Medical University, Beijing, China; 8 Department of Laboratory Medicine, Harbin Medical University Cancer Hospital, Harbin, Heilongjiang, China; University of Texas MD Anderson Cancer Center, United States of America

## Abstract

**Background:**

Excision repair cross-complimentary group 1 (ERCC1) is an essential component of the nucleotide excision repair system that is responsible for repairing damaged DNA. Functional genetic variations in the *ERCC1* gene may alter DNA repair capacity and modulate cancer risk. The putative roles of *ERCC1* gene polymorphisms in lung cancer susceptibility have been widely investigated. However, the results remain controversial.

**Objectives:**

An updated meta-analysis was conducted to explore whether lung cancer risk could be attributed to the following *ERCC1* polymorphisms: rs11615 (T>C), rs3212986 (C>A), rs3212961 (A>C), rs3212948 (G>C), rs2298881 (C>A).

**Methods:**

Several major databases (MEDLINE, EMBASE and Scopus) and the Chinese Biomedical database were searched for eligible studies. Crude odds ratios (ORs) with 95% confidence intervals (CIs) were used to measure the strength of associations.

**Results:**

Sixteen studies with 10,106 cases and 13,238 controls were included in this meta-analysis. Pooled ORs from 11 eligible studies (8,215 cases vs. 11,402 controls) suggested a significant association of *ERCC1* rs11615 with increased risk for lung cancer (homozygous: CC versus TT, OR = 1.24, 95% CI: 1.04–1.48, *P* = 0.02). However, such an association was disproportionately driven by a single study. Removal of that study led to null association. Moreover, initial analyses suggested that *ERCC1* rs11615 exerts a more profound effect on the susceptibility of non-smokers to lung cancer than that of smokers. Moreover, no statistically significant association was found between remaining *ERCC1* polymorphisms of interest and lung cancer risk, except for rs3212948 variation (heterozygous: CG vs.GG, OR = 0.78, 95% CI: 0.67–0.90, *P* = 0.001; dominant: CG/CC vs.GG, OR = 0.79, 95% CI: 0.69–0.91, *P = *0.001).

**Conclusion:**

Overall, this meta-analysis suggests that *ERCC1* rs3212948 G>C, but not others, is a lung cancer risk-associated polymorphism. Carefully designed studies with large sample size involving different ethnicity, smoking status, and cancer types are needed to validate these findings.

## Introduction

It has become clear that the susceptibility to disease varies from one individual to another. Some heritable characteristics that shape the effects of environmental exposure may contribute to the variable susceptibilities among people. Smoking highly increases risk of lung cancer by up to 20 folds since many carcinogens in cigarette smoke can be converted into reactive metabolites in human body. These reactive products (e.g., diol epoxide derivatives of polycyclic aromatic hydrocarbons) can potentially damage cellular DNA and cause the formation of DNA adducts via covalent binding or oxidation. The resulting adducts are carcinogenic and may block the transcription of critical genes or result in mutations at hot spots [1]. Nevertheless, only a proportion of cigarette smokers and victims of second-hand smoke develop lung cancer in their lifetime, which may be partially attributed to the fact that DNA repair systems (e.g., nucleotide excision repair) can effectively remove DNA lesion and restore genomic integrity. It has been suggested that DNA repair capacity is essential in protecting people from cigarette smoke-related carcinogenesis [2], [3]. The nucleotide excision repair (NER) system is responsible for the repair of various DNA lesions, such as bulky adducts, cross links, oxidative DNA damage, thymidine dimers and alkylating damage. Genetic variations in DNA repair genes may alter DNA repair capacity and modulate cancer risk in the host. Excision repair cross-complimentary group 1 (ERCC1) is a critical protein in the NER pathway. Typically, ERCC1 joins XPF endonuclease (also known as ERCC4) to form heterodimeric endonuclease (XPF-ERCC1), which excises the 5′ end of DNA to the damaged site. XPF-ERCC1 complex also participates in homologous recombination and the repair of inter-strand crosslinks. Thus, functional polymorphisms in the *ERCC1* gene that compromise DNA repair capacity may be a potential risk factor for tobacco-induced cancers. Therefore, potential associations between *ERCC1* gene polymorphisms and cancer risk have evoked great interest. Due to lack of non-synonymous single nucleotide polymorphism (SNP) in the coding region of the *ERCC1* gene, most studies have focused on the rs3212986 (3′UTR C8092A) and synonymous rs11615 (exon 4 T19007C) polymorphisms, which are believed to influence transcript stability and mRNA levels [4], [5], respectively. Numerous studies on such topics have been conducted over the past decades, but the results remain controversial. Several meta-analyses have also yielded conflicting conclusions [6]–[8]. Lately, more case-control studies regarding these topics have emerged. Apart from those two common *ERCC1* variants, associations of three other *ERCC1* polymorphisms (rs3212961 (17677A>C), rs3212948 G>C, and rs2298881 C>A) with lung cancer risk have also gained increasing attentions. Therefore, we conducted this updated meta-analysis to reassess the associations of the first two common polymorphisms in the *ERCC1* gene and lung cancer risk, and explore the influence of the other three polymorphisms on predisposition to lung cancer.

## Materials and Methods

### Publication search

A systematic literature search throughout the MEDLINE, EMBASE and Scopus databases was performed with the use of the following search terms: “*ERCC1* or excision repair cross-complimentary group 1”, “DNA repair”, “polymorphism or variant or variation” and “lung cancer or tumor or carcinoma or neoplasm”. Publications written in Chinese were also searched from the Chinese Biomedical (CBM) database (http://cbmwww.imicams.ae.cn/cbmbin) (1978–) to increase the coverage of our current study using the combination of terminologies: “*ERCC1*”, “polymorphism” and “lung cancer” in Chinese. In addition, references of the retrieved research or review articles on this topic were manually reviewed to identify extra eligible studies. Publication search was initiated on October 12, 2013, and last search was performed on November 15, 2013.

### Selection of eligible studies

Selected studies must meet the following inclusion criteria: 1) studies investigating any type or mixed types of lung cancer (small cell lung cancer or non- small cell lung cancer (NSCLC): adenocarcinoma, squamous carcinoma, and large-cell carcinoma, etc.), regardless of smoking status; 2) original research studies written in English or Chinese, 3) case-control, nested case-control, or cohort study, and 4) adequate information to calculate odds ratio (OR) and 95% confidence interval (CI). Exclusion criteria were: 1) duplicate data, 2) abstract, case report, comment, review, and editorial, 3) lack of sufficient genotyping data, and 4) studies involving subjects with family history or cancer-prone disposition. In the case of studies with overlapping subjects, the latest and/or largest study was chosen. Studies in which the genotype frequencies of the control group departed from Hardy-Weinberg equilibrium (HWE) was excluded from the final analysis, unless there was further evidence validating HWE from another *ERCC1* polymorphisms.

### Data Extraction

Information extraction was conducted individually by two investigators (Zhu JH and Hua RX) from each eligible study. The main information comprises first author, year of publication, country of origin, ethnicity, cancer type, source of controls (i.e., population-based or hospital-based), genotyping method, number of cases and controls, genotype counts of five *ERCC1* polymorphisms for cases and controls, and main findings. In the case that studies included subjects of different ethnic groups, data were obtained separately for each ethnic group and labeled as Caucasian or Asian.

### *ERCC1* gene expression analysis based on *ERCC1* variant genotypes

The biological plausibility of our findings was investigated by correlating respective *ERCC1* polymorphism genotypes and corresponding *ERCC1* mRNA expression levels in 270 lymphoblastoid cell lines. Genotype data of *ERCC1* polymorphisms and *ERCC1* mRNA expression information were retrieved from HapMap website (http://www.hapmap.org) and SNPexp online tool (http://app3.titan.uio.no/biotools/help.php?app=snpexp), respectively. These resources facilitate researchers to determine the correlation of HapMap genotypes in a genomic region of interest and gene expression levels [9]. The international HapMap phase (II+III) release #28 data set contain genotype data of 3.96 million polymorphisms for 270 individuals from four worldwide populations [CEU: 90 Utah residents with ancestry from northern and western Europe; CHB: 45 unrelated Han Chinese in Beijing; JPT: 45 unrelated Japanese in Tokyo; YRI: 90 Yoruba in Ibadan, Nigeria] [10]. The mRNA expression data were obtained from the same 270 individuals (GENe Expression VARiation, http://www.sanger. ac.uk/resources/software/genevar/).

### Statistical Methods

Crude odds ratio (OR) with 95% confidence interval (CI) were used to access the association between each of the five *ERCC1* polymorphisms and lung cancer risk. Letters V and W represented variant and wild alleles of each polymorphism. Different genetic models were adopted during risk estimation: homozygous (VV vs. WW), heterozygous (WV vs. WW), recessive (VV vs. WV/WW), and dominant (VV/WV vs. WW) model. A chi-square-based *Q*-test was used to check heterogeneity assumption. A *P-*value of >0.10 for the Q test suggested the absence of heterogeneity across the studies, and then the fixed-effects model (the Mantel–Haenszel method) [11] would be adopted; otherwise, the random-effects model (the DerSimonian and Laird method) [12] would be performed. The Z-test was used to determine the significance of the pooled OR. The funnel plot was created to test publication bias. Briefly, the standard error of log (OR) of each investigation was plotted against its log (OR), and the asymmetry of funnel plot was assessed by the method of Egger's linear regression test [13]. Furthermore, sensitivity analysis was used to determine stability of the result, i.e., an individual study was excluded at a time, and then risk estimates were recalculated to examine the effect of single study on the pooled ORs. Hardy-Weinberg equilibrium (HWE) in the control group for each *ERCC1* polymorphism was checked by the Pearson's goodness-of-fit chi-square test. Student's t test and analysis of variance test were used to evaluate the differences in the relative mRNA expression levels among different genotype groups. All analyses were performed by using STATA version 11.0 (Stata Corporation, College Station, TX) and SAS version 9.1 (SAS Institute, Cary, NC). All statistical analyses were two-sided, and *P*<0.05 was considered significant.

## Results

### Characteristics of studies

**Figure 1:**
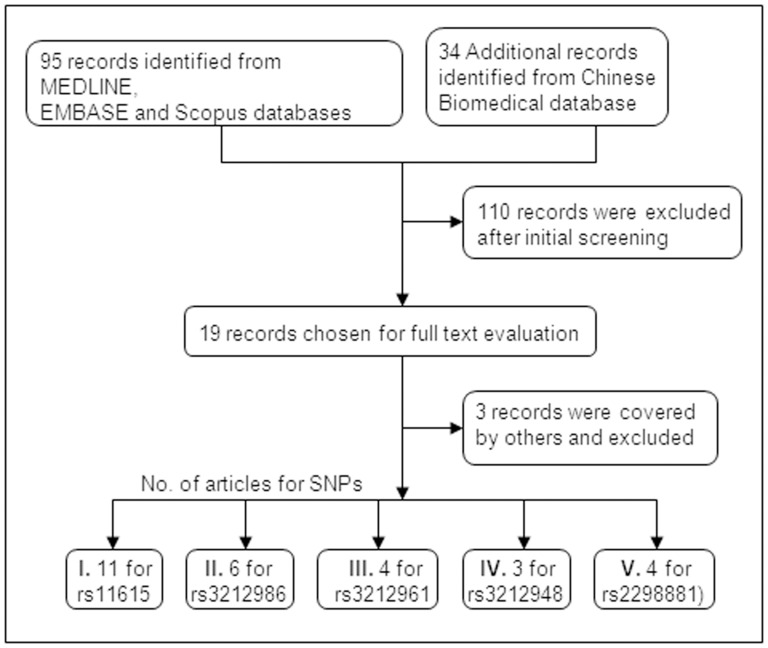
Flow diagram of included studies for the associations between *ERCC1* polymorphisms and lung cancer risk.

Literature search initially produced 129 articles. After screening and reviewing articles for study eligibility, 113 articles were excluded due to review articles or non case-control studies, overlapped participants, failure to report genotype distribution data, and other reasons described previously [14] (Figure 1). For example, among the 15 studies concerning *ERCC1* rs11615 polymorphism [4], [15]–[28], 5 were reported by Yin JY *et al*.[18], [25]–[28], in which samples were collected in the same institute, Liaoning Cancer Hospital. One of them [25] was not a case-control study and thus was excluded. In order to avoid repetition in the sampling, we chose one [18] from the rest of 4 studies [18], [26]–[28], which has the largest sample size and also was published most lately. Ultimately, 16 studies [4], [5], [15]–[24], [28]–[31] with 10,106 cases and 13,238 controls were chosen for this meta-analysis (Figure 1). Of the resulting 16 studies, 9 were conducted in Chinese population, and 7 in Caucasian population. All studies reported the association between at least one *ERCC1* polymorphism of interest and lung cancer risk. Studies, inspecting the associations between multiple *ERCC1* genetic variations and lung cancer risk were divided into several sub-studies, each of which encompassed the analysis of a single polymorphism. Genotyping in these 16 studies was performed via different methods, details of which were listed in Table 1. Overall, the current meta-analysis contained 11 studies for rs11615 [4], [15]–[24], 6 for rs3212986 [4], [15], [16], [20], [22], [31], 4 for rs3212961 [4], [16], [28], [30], 3 for rs3212948 [5], [29], [30], and 4 for rs2298881 [20], [22], [28], [29] polymorphisms, respectively. Most of these studies concerned all types of lung cancer among both smokers and non-smokers, except for two. One study conducted by Zienolddiny *et al*. [4] focused on NSCLC in smokers and former smokers, while the other by Yin *et al*. [17] investigated only adenocarcinoma in non-smokers.

**Table 1:** Characteristics of studies that explored the association between *ERCC1* polymorphisms and lung cancer risk.

Surname	Year	Histology	Country	Ethnicity	Control	Genotyping	Polymorphisms	HWE	Case	Control
					source	methods				
Vogel	2004	mixed*	Denmark	Caucasian	PB	Real-time PCR	rs11615 T>C	0.313	252	266
Zhou	2005	mixed	USA	Caucasian	PB	TaqMan	rs11615 T>C	0.541	1752	1358
							rs3212986 C>A	0.985		
Shen	2005	mixed	China	Asian	PB	real-time PCR	rs3212948 G>C	0.752	118	112
							rs3212961 A>C	0.760		
Matullo	2006	mixed	European	Caucasian	PB	TaqMan	rs11615 T>C	0.156	116	1093
Zienolddiny	2006	NSCLC	Norway	Caucasian	PB	TaqMan	rs11615 T>C	0.670	260	213
							rs3212986 C>A	0.088		
							rs3212961A>C	0.025		
Ma	2007	mixed	China	Chinese	HB	TaqMan	rs2298881 C>A	0.190	992	986
							rs3212948 G>C	0.171		
Yu	2008	mixed	China	Chinese	PB	PCR-RFLP	rs11615 T>C	0.524	988	986
							rs3212986 C>A	0.335		
							rs3212961 A>C	0.515		
Zhang	2008	mixed	China	Chinese	HB	TaqMan	rs11615 T>C	0.988	291	273
Yin	2009	AC	China	Chinese	HB	PCR-RFLP	rs11615 T>C	0.980	285	285
Deng	2011	mixed	China	Chinese	PB	Real-time PCR	rs11615 T>C	0.743	315	313
Jones	2011	mixed	USA	Caucasian	PB	TaqMan	rs3212948 G>C	0.848	427	737
Yin	2011		China	Chinese	HB	LDR-PCR	rs11615 T>C	0.400	330	335
							rs2298881 C>A	0.682		
							rs3212961 A>C	0.338		
Kang	2011	mixed	China	Chinese	HB	PCR-RFLP	rs3212986 C>A	0.018	200	200
Sakoda	2012	mixed	USA	Caucasian	PB	TaqMan	rs11615 T>C	0.013	744	1477
							rs3212986 C>A	0.122		
							rs2298881 C>A	0.350		
Yin	2012	mixed	China	Chinese	HB	PCR-RFLP	rs11615 T >C	0.663	357	378
Kazma	2012	mixed	European	Caucasian	HB	HumanHap300	rs11615 T >C	0.185	2679	4226
						BeadChips				
							rs3212986 C>A	0.732		
							rs2298881 C>A	0.787		

AC, adenocarcinoma; HB, Hospital based; NSCLC, non small cell lung cancer; PB, Population based; PCR-RFLP, Polymerase chain reaction-restriction fragment length polymorphism; LDR-PCR, ligase detection reaction coupled with PCR; MAF, Minor allele frequency; HWE, Hardy-Weinberg equilibrium.

*mixed: small cell lung cancer, non-small cell lung cancer (adenocarcinoma, squamous carcinoma, large-cell carcinoma, etc.)

### *ERCC1* rs11615 (T>C) polymorphism

**Figure 2:**
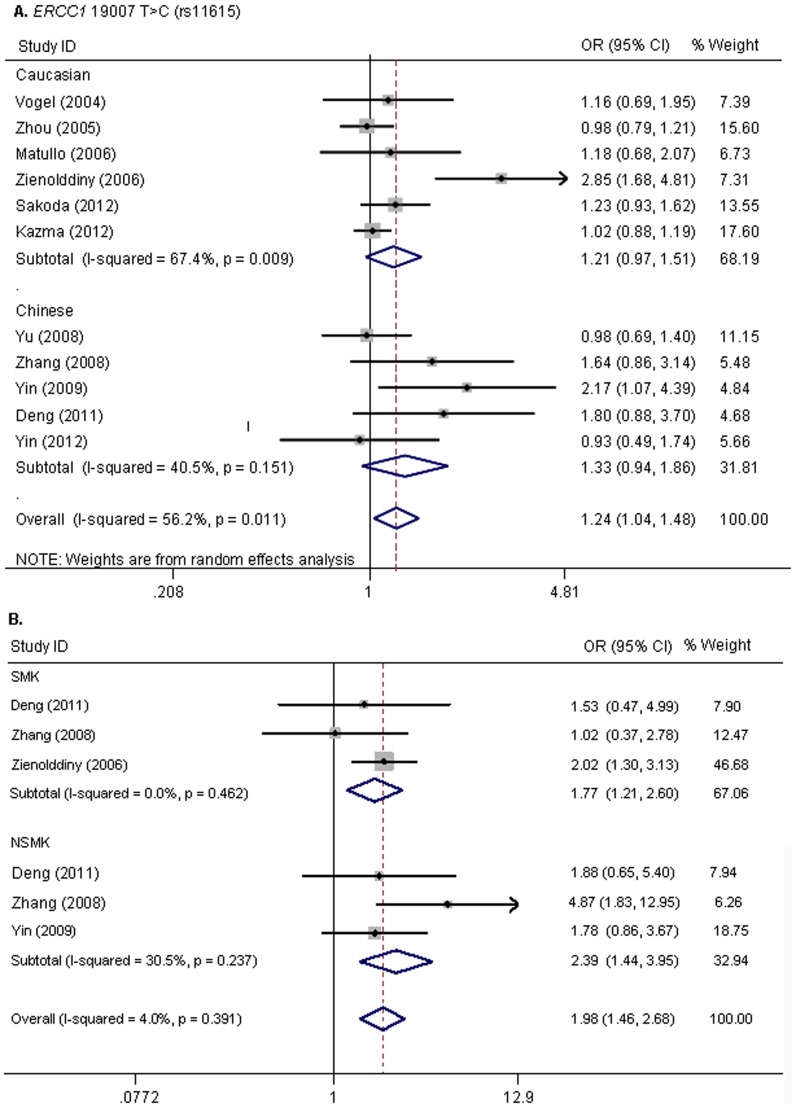
Forest plots of lung cancer risk associated with the *ERCC1* polymorphism. A, Forest plot of risk of lung cancer associated with the *ERCC1* rs11615 polymorphism by a homozygous model. B,Forest plot of lung cancer risk associated with the *ERCC1* rs11615 polymorphism in the stratified analyses by smoking status. The plots of heterozygous model were shown. SMK, smoker; NSMK, non-smoker. The estimate of OR and its 95% CI are plotted with a box and a horizontal line for each study; ◊ represents pooled ORs and its 95% CIs.

In the meta-analysis of 11 studies with 8,215 cases and 11,402 controls, the pooled OR for the association between *ERCC1* rs11615 polymorphism and lung cancer risk was statistically significant (homozygous: CC versus TT, OR = 1.24, 95% CI: 1.04–1.48, *P* = 0.02) with moderate among-study heterogeneity (*I^2^* = 22.8%, *P* = 0.01) (Figure 2A). Stratified analysis by ethnicity or source of control detected no notable associations among any subgroup (i.e., Asian versus Caucasian populations, hospital-based versus population-based). We then removed one study at a time to explore the source of heterogeneity. It was found that exclusion of the study by Zienolddiny *et al*. [4] reduced the *I^2^* value to 11.3% (*P* = 0.338), and then significance of the association no longer existed (Table 2). This study represented only 7.31% weight of the meta-analysis. These results suggested that this study dictates the among-study heterogeneity, and drives the initially observed risk association. While carefully reviewing Zienolddiny's study, it was found that this study was undertaken among smokers. Subjects in both case and control group were either current smokers or ex-smokers who have quit smoking for less than 5 years. In particular, mean cigarettes per day for years of smoking were 15.6 ± 8.3 and 14.8± 6.3 for 40.4 ±12.1 and 42.3 ±7.9 years in cases and controls, respectively. These inclusion criteria led to a minor allele frequency (MAF) of 0.46 in this study that was deviated from MAFs of around 0.60 in Caucasian population. Therefore, the recruit of subjects with long smoking history might be the main factor accounting for the heterogeneity.

**Table 2:** Results of overall and stratified analyses for the associations of *ERCC1* polymorphisms and risk of lung cancer**.

Variables	No. of	Sample size	Homozygous			Heterozygous			Dominant			Recessive		
	studies	Case/control	OR (95% CI)	*P^het^*	I*^2^* (%)	OR (95% CI)	*P^het^*	I*^2^* (%)	OR (95% CI)	*P^het^*	I*^2^* (%)	OR (95% CI)	*P^het^*	I*^2^* (%)
**rs11615 T>C** **														
Total	10	7779/10655	1.08 (0.98–1.19)	0.338	11.3	1.02 (0.95–1.09)	0.391	5.4	1.03 (0.96–1.10)	0.379	6.8	1.05 (0.97–1.13)	0.361	8.8
Ethnicities														
Asian	5	2236/2235	1.24 (0.97–1.58)	0.151	40.5	1.23 (0.96–1.58)	0295	18.9	1.24 (0.98–1.57)	0.191	34.6	1.05 (0.93–1.18)	0.179	36.3
Caucasian	5	5543/8420	1.05 (0.95–1.17)	0.723	0.0	1.00 (0.93–1.08)	0.697	0.0	1.01 (0.94–1.09)	0.883	0.0	1.05 (0.95–1.16)	0.465	0.0
Source of control														
HB	4	3612/5162	1.08 (0.94–1.24)	0.107	50.7	1.05 (0.95–1.16)	0.108	50.6	1.05 (0.95–1.15)	0.09	53.6	1.02 (0.91–1.14)	0.326	13.3
PB	6	4167/5493	1.09 (0.95–1.25)	0.543	0.0	0.99 (0.89–1.09)	0.726	0.0	1.01 (0.92–1.12)	0.70	0.0	1.08 (0.97–1.20)	0.319	14.8
Smoking status														
Smokers	3	657/468	**2.16 (1.41–3.30)**	0.099	56.8	**1.77 (1.21–2.60)**	0.462	0.0	**1.94 (1.36–2.79)**	0.234	31.2	1.32 (0.83–2.09)	0.042	68.6
Non-smokers	3	494/616	**2.39 (1.47–3.88)**	0.542	0.0	**2.39 (1.44–3.95)**	0.237	30.5	**2.40 (1.49–3.85)**	0.399	0.0	1.19 (0.94–1.52)	0.628	0.0
**rs3212986 C>A**														
Total	6	6639/8630	1.00 (0.88–1.14)	0.609	0.0	0.97 (0.91–1.04)	0.737	0.0	0.97 (0.91–1.04)	0.784	0.0	1.01 (0.89–1.15)	0.558	0.0
Ethnicities														
Asian	2	1188/1186	1.05 (0.80–1.37)	0.905	0.0	1.00 (0.84–1.20)	0.950	0.0	1.01 (0.86–1.20)	0.939	0.0	1.05 (0.83–1.33)	0.846	0.0
Caucasian	4	5451/7444	0.98 (0.85–1.14)	0.330	12.6	0.96 (0.89–1.04)	0.463	0.0	0.97 (0.90–1.04)	0.539	0.0	1.00 (0.86–1.15)	0.287	20.4
Source of control														
HB	2	2836/4428	1.00 (0.82–1.21)	0.770	0.0	0.91 (0.83–1.01)	0.777	0.0	0.93 (0.84–1.02)	0.662	0.0	1.04 (0.86–1.24)	0794	0.0
PB	4	3803/4202	1.00 (.83–1.19)	0.319	14.6	1.02 (0.93–1.12)	0.969	0.0	1.02 (0.93–1.11)	0.949	0.0	0.99 (0.83–1.18)	0.288	20.2
**rs3212961 A>C**														
Total	4	1770/1830	0.87 (0.71–1.07)	0.318	14.8	0.92 (0.77–1.11)	0.216	32.7	0.90 (0.76–1.07)	0.167	40.8	0.91 (0.79–1.06)	0.855	0.0
Ethnicities														
Asian	3	1436/1428	0.87 (0.71–1.08)	0.177	42.3	0.92 (0.76–1.11)	0.108	55.1	0.91 (0.76–1.08)	0.082	60.1	0.93 (0.79–1.09)	0.749	0.0
Caucasian	1	334/402	0.79(0.36–1.74)			0.92 (0.40–2.10)			0.82 (0.38–1.80)			0.85 (0.61–1.19)		
**rs3212948 G>C**														
Total	3	1537/1835	0.84 (0.65–1.08)	0.643	0.0	**0.78 (0.67–0.90)**	0.321	12.0	**0.79 (0.69–0.90)**	0.287	19.9	0.93 (0.73–1.19)	0.782	0.0
**rs2298881 C>A**														
Total	4	4653/6921	1.11 (0.91–1.36)	0.938	0.0	1.03 (0.94–1.13)	0.530	0.0	1.04 (0.95–1.13)	0.530	0.0	1.10 (0.91–1.32)	0.964	0.0
Ethnicities														
Asian	2	1317/1329	1.13 (0.90–1.43)	0.562	0.0	1.04 (0.88–1.22)	0.732	0.0	1.06 (0.90–1.24)	0.623	0.0	1.11 (0.90–1.37)	0.634	0.0
Caucasian	2	3336/5592	1.06 (0.73–1.56)	0.992	0.0	1.03 (0.92–1.15)	0.148	52.1	1.03 (0.93–1.14)	0.168	47.3	1.06 (0.72–1.55)	0.926	0.0

CI, confidence interval; OR, odds ratio. The results were in bold, if the 95% CI excluded 1 or *P*<0.05.

*P* ^het^ value of Q-test for heterogeneity test; random-effects model was used when *P*<0.05 for heterogeneity test; otherwise, fixed-effects model was used. Significant results are listed in bold.

**Study by Zienolddiny *et al*. was excluded for rs11615 analysis.

Furthermore, four of eleven publications including six studies (1,151 cases and 1,084 controls) reported genotype distributions of rs11615 polymorphism for smokers and non-smokers. With these extra information based on smoking status, stratified analyses by smoking status showed that the association of the rs11615 polymorphism with lung cancer risk was stronger in non-smokers than in smokers (homozygous, OR (95% CI): 2.39 (1.47–3.88)/2.16 (1.41–3.30); heterozygous, 2.39 (1.44–3.95)/1.77(1.21–2.60); dominant, 2.40 (1.49–3.85)/1.94 (1.36–2.79)) without substantial heterogeneity (Figure 2B,Table 2).

### *ERCC1* rs3212986 C>A polymorphism

**Figure 3:**
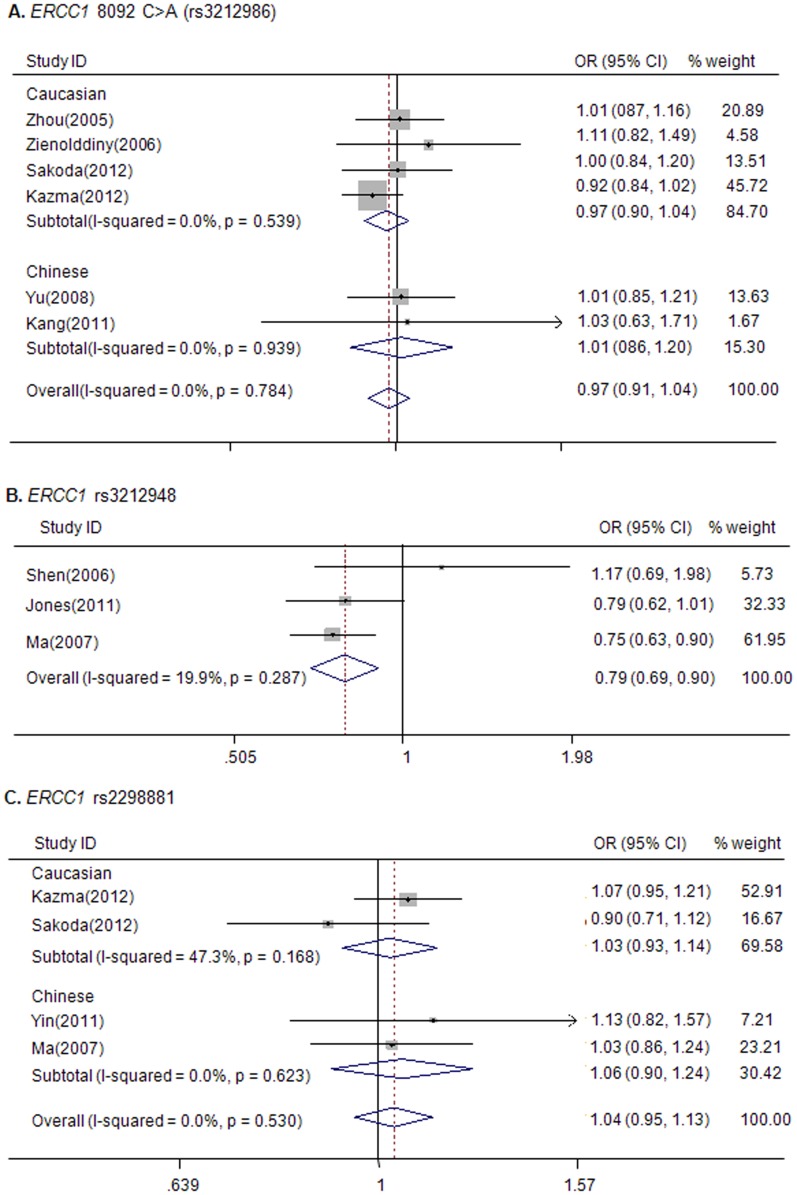
Forest plots of lung cancer risk associated with the *ERCC1* polymorphisms. A, Forest plot of lung cancer risk associated with the *ERCC1* rs3212986 polymorphism. The plot of dominant model was shown. B, Forest plot of lung cancer risk associated with the *ERCC1* rs3212948 polymorphism. The plot of dominant model was shown. C, Forest plot of lung cancer risk associated with the *ERCC1* rs2298881 polymorphism. The plot of dominant model was shown.

To investigate potential associations of *ERCC1* rs3212986 polymorphism with risks for lung cancer, 6 eligible studies with 6,639 cases and 8,630 controls were pooled together for analysis [4], [15], [16], [20], [22], [31]. No significant association was found (homozygous, AA versus CC, OR = 1.00, 95% CI: 0.88–1.14; heterozygous, CA versus CC, OR = 0.97, 95% CI: 0.91–1.04; dominant, AA/CA versus CC, OR = 0.97, 95% CI: 0.91–1.04, recessive, AA versus CC/CA, OR = 1.01, 95% CI: 0.89–1.15) (Figure 3A,Table 2). In the study by Kang *et al*. [31], genotype distribution in the control group did not follow HWE. This study was included in the final analysis because removing it did not qualitatively alter the association. Moreover, no significant association was revealed in either the Asian or the Caucasian subgroup (Table 2).

### *ERCC1* rs3212961 A>C polymorphism

To date, four eligible studies investigating the roles of *ERCC1* rs3212961 in the lung cancer risk were available [4], [16], [28], [30]. These studies encompassed 1,770 cases and 1,830 controls. Pooled analysis failed to provide statistical evidence for a significant association of *ERCC1* rs3212961 polymorphism with overall lung cancer risk (homozygous: CC versus AA, OR = 0.87, 95% CIs: 0.71–1.07; heterozygous: CA versus AA, OR = 0.92, 95% CIs:0.77–1.11, dominant, CA/CC versus AA, OR = 0.90, 95% CIs: 0.76–1.07, and recessive model, CC versus AA/CA 0.91 95% CIs: 0.79–1.06) without substantial between-study heterogeneity(Table 2). Owing to the relative small number and minor heterogeneity of the available studies, sensitivity analysis on this polymorphism was not performed.

### *ERCC1* 3212948 G>C polymorphism

A tagSNP *ERCC1* 3212948 G>C, representing common genetic variation in the coding region of the *ERCC1* gene [5], has increasingly received attention. Three eligible studies with 1,537 cases and 1,835 controls were identified [5], [29], [30]. This variation was found to be significantly associated with decreased lung cancer risk (heterozygous: CG versus GG, OR = 0.78, 95% CI: 0.67–0.90, *P* = 0.001; dominant: CG/CC versus GG, OR = 0.79, 95% CI: 0.69–0.91, *P = *0.001) without obvious heterogeneity (Figure 3B,Table 2).

### *ERCC1* rs2298881 C>A polymorphism

Four eligible studies with 4,653 cases and 6,921 controls were acquired for evaluating the association of another *ERCC1* tagSNP rs2298881 with lung cancer [20], [22], [28], [29]. The overall pooled ORs were calculated as indicated below: homozygous: AA versus CC OR = 1.11, 95% CI: 0.91–1.36, *P* = 0.288; heterozygous: OR = 1.03, 95% CI: 0.94–1.13, *P* = 0.53; dominant: AC/AA versus CC, OR = 1.04, 95% CI: 0.95–1.13), *P* = 0.41; recessive: OR = 1.10, 95% CI: 0.91–1.32, *P* = 0.322. Risk estimates suggested that *ERCC1* rs2298881 is not a risk-associated polymorphism in lung cancer (Figure 3C, Table 2).

### Sensitivity analyses

As mentioned earlier, the study by Zienolddiny *et al*. [4] was removed from the final analysis for *ERCC1* rs11615 polymorphism. Subsequently, influence analysis showed that omitting any other study essentially did not alter the results, thereby confirming the stability of this meta-analysis. Next, the same analysis was performed for *ERCC1* rs3212986 polymorphism, and the association remained unchanged, suggesting that this pooled analysis is also stable.

### Publication bias

**Figure 4:**
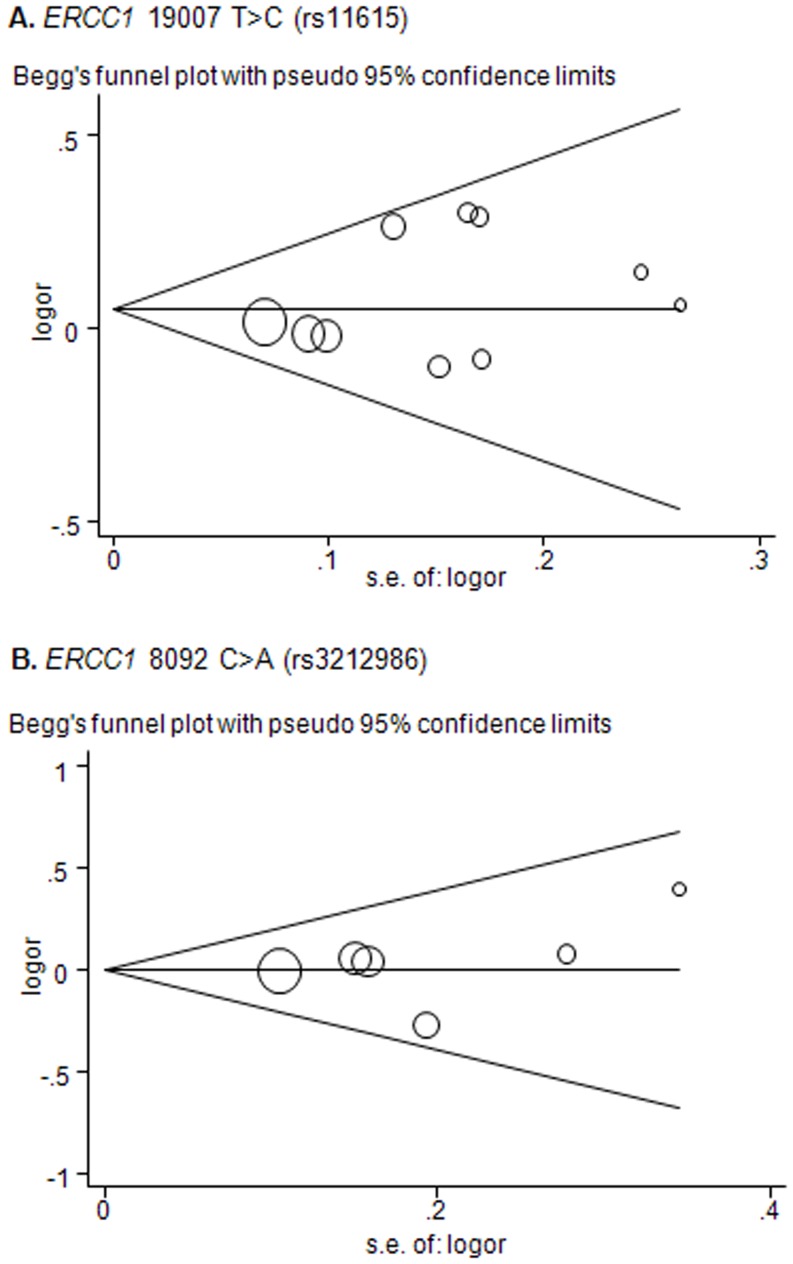
Funnel plots of Begg's were used to detect publication bias on *ERCC1* rs11615 (recessive model) and *ERCC1* rs3212986 polymorphisms (homozygous model). No significant publication bias was found. Each point represents a separate study for the indicated association. Size of each point is proportional to its weight.

Begg's funnel plot and Egger's test were conducted to detect the publication bias of the meta-analysis. The shapes of the funnel plot for the *ERCC1* rs11615 polymorphism seemed symmetrical under all models (Figure 4). Nonetheless, Egger's test under homozygous and dominant models were significant (*P* = 0.03 for CC versus TT, *P* = 0.03 for TC/CC versus TT, *P* = 0.06 for TC vs. TT, *P* = 0.28 for CC vs. TC/TT). The publication bias did not alter conclusion, because results in all comparison models unequivocally suggested that the pooled OR for the association was not statistically significant. Furthermore, no publication bias was detected for associations of rs3212986 polymorphism with lung cancer risk (Figure 4), which was confirmed by Egger's test (*P* = 0.55 for AA vs. CC, *P* = 0.16 for CA vs. CC, *P* = 0.08 for CA/AA vs. CC, and *P* = 0.73 for AA vs. AC/CC).

### Correlation between genotypes of *ERCC1* polymorphisms and mRNA expression

**Figure 5:**
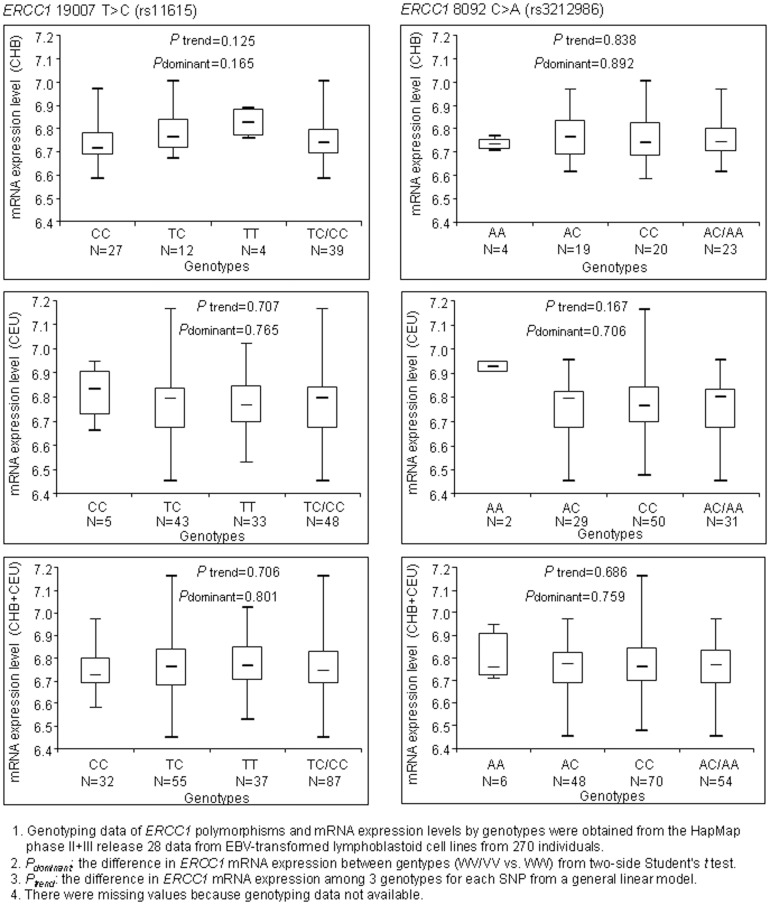
*ERCC1* mRNA expression by the genotypes of *ERCC1* rs11615 and rs3212986 polymorphisms.

It was thought that *ERCC1* rs3212986 and rs11615 may affect transcript stability and mRNA levels, respectively [4], [5]. However, our results did not reveal any significant association between either of polymorphisms and lung cancer risk. As previously published [14], [32], we used information from HapMap and SNPexp online tool to further estimate whether expression levels of *ERCC1* transcript correlate with genotypes of these two polymorphisms. Given subjects in our meta-analysis were from Chinese and Caucasian populations, we performed the correlation analyses for CHB (Chinese), CEU (Caucasian), and a combination of these two populations, respectively. Information on genotype of rs11615 polymorphism and corresponding *ERCC1* mRNA expression was available for 32 CC (GG), 55 TC (AG), and 37 TT (AA) individuals. For *ERCC1* rs3212986 polymorphism, there were 6 AA, 48 AC, and 70 CC carriers with expression data (Figure 5). Although the expression levels of *ERCC1* transcript showed a trend of decreasing from wide type (TT) to the homozygous variant (CC) genotype of rs11615 polymorphism in CHB population, the difference did not reach statistical significance. Overall, there was no significant difference in *ERCC1* transcript expression levels among different genotypes of rs11615 polymorphism in CHB, CEU, and combined populations under all the genetic models. Similarly, genotype of *ERCC1* rs3212986 polymorphism did not appear to correlate with mRNA expression level of *ERCC1* gene, either. The effect of *ERCC1* rs3212948 polymorphism in *ERCC1* gene expression was also explored since it was suggested to associate with decreased lung cancer risk. Results indicated that rs3212948 was related to decreased expression levels of the *ERCC1* gene in CHB, but increased expression in CEU. (recessive model: CEU: *P* = 0.0433; CHB: *P* = 0.0538) (Figure S1).

## Discussion

Accumulating studies of the associations between *ERCC1* polymorphisms and lung cancer risk have been conducted, but yielded conflicting results. The current meta-analysis examined whether five most commonly studied *ERCC1* polymorphisms (rs11615, rs3212986, rs3212961, rs3212948, and rs2298881) were associated with lung cancer risk. Association between *ERCC1* rs11615 or rs3212986 polymorphism and risk of lung cancer is seemingly biologically worthy of approval, since these two polymorphisms have been thought to alter transcript stability and mRNA levels [4], [5], respectively. Nevertheless, results drawn from the current meta-analysis did not support such an association. Initially, the meta-analysis indicated a significant association of the *ERCC1* rs11615 polymorphism with lung cancer under the homozygous model with moderate corresponding among-study heterogeneity (*I^2^* = 22.8%, *P* = 0.01). Nonetheless, influence analysis unveiled that the association mainly arose from one study by Zienolddiny and colleagues [4], which represented only 7.31% weight in the whole meta-analysis. Moreover, this meta-analysis indicates no statistically significant association between *ERCC1* rs3212986 polymorphism and lung cancer risk.

Recently, several meta-analyses have reported conflicting conclusion regarding to the association between the *ERCC1* rs11615 polymorphism and lung cancer risk [6]–[8]. Initially, a stratified analysis involving 4 studies of 2,279 cases and 2,808 controls in a meta-analysis suggested no association between this polymorphism and lung cancer risk [7]. Cao *et al*. analyzed total 7 studies [4], [15], [16], [19], [21], [24], [26] with 3,810 cases/4,332 controls and found similar results. Recently, Zhang *et al*. showed conflicting evidence, whose meta-analysis focusing on the contribution of *ERCC1* polymorphisms on overall cancer risk. Stratified analysis with 9 studies [4], [15]–[17], [19], [21], [23], [24], [26], [27] of 4,652 cases and 5,164 controls demonstrated that *ERCC1* rs11615, but not rs3212986 polymorphism significantly increased lung cancer risk.

Compared to these previous meta-analyses, the current study has several unique features regarding rs11615 polymorphism. First, three new studies [18], [20], [22] published in 2012 containing 3,780 cases and 6,081 controls were included in the current analysis. Second, two studies [26], [27] that were incorporated in Zhang's meta-analysis were excluded from the current analysis [6]. During intensive literature review, four articles conducted by Yin *et al*. [18], [26]–[28] in the same hospital appeared to fit selection criterion. To eliminate the possibility that samples might be repeatedly used in these studies, we ended up keeping only one study, which had the largest sample size, and was published most lately. Third, the study by Zienolddiny *et al*. [4] was removed from the final analysis due to its disproportional contribution to the risk estimates and among-heterogeneity. Fourth, available data were stratified and analyzed based on the smoking status of study participants. Our preliminary result suggested that the association between *ERCC1* rs11615 and lung cancer risk might be stronger in non-smokers than in smokers, which may be in accordance with the concept of genetic susceptibility that indicate individuals with predisposition to cancer tend to develop cancer when suffering low-dose hazardous exposure (e.g., second-hand smoke). However, due to relatively small sample size and selection bias, this result should be interpreted with caution. Fifth, when compared to the latest meta-analysis concerning *ERCC1* rs11615 polymorphism [6], samples size in this meta-analysis almost doubled. Moreover, In accordance with previous meta-analyses [6], [8], this analysis confirmed that rs3212986 polymorphism applies null effect on lung cancer risk. Furthermore, it was found that correlations of *ERCC1* rs11615 or rs3212986 polymorphism with corresponding *ERCC1* mRNA expression levels were negative. These results provide evidence supporting our findings that these two *ERCC1* polymorphisms were not associated with lung cancer susceptibility.

*ERCC1* rs3212961 polymorphism has been also well characterized. With 4 studies (1,770 cases and 1,830 controls), this meta-analysis demonstrated no association between this polymorphism and lung cancer risk. We also evaluated two additional *ERCC1* tagging SNPs (rs3212948 and rs2298881). Risk estimates suggested that *ERCC1* rs3212948 is a protection-associated genetic variation in lung cancer, whereas no association was found for rs2298881. To our knowledge, this is the first meta-analysis that estimated associations of *ERCC1* rs3212961, rs3212948 and rs2298881 polymorphisms with lung cancer risk. Nevertheless, correlation analysis between genotypes of rs3212948 polymorphisms and mRNA expression did not agree with association study. Results for correlation suggested that rs3212948 might decrease expression levels of the *ERCC1* gene in CHB, but increase the expression in CEU (recessive model: CEU: *P* = 0.0433; CHB: *P* = 0.0538). Overall, no significant correlation was found in combined CHB and CEU population. Several reasons may help to explain the discrepancy: 1) the number of available studies for rs3212948 was very few, and meta-analyses with more studies are needed to validate our findings on association. 2) with only three studies, we were unable to perform efficient stratification analysis by ethnicity to investigate the association between rs3212948 and lung cancer risk in either Chinese or Caucasian group. However, the results convey some important information: 1) *ERCC1* rs3212948 polymorphism may influence mRNA expression and 2) the effect on mRNA expression is ethnicity-dependent. These findings may promote researchers to investigate biological function of *ERCC1* rs3212948 polymorphism.

There are some limitations in the current up-to-date meta-analyses. First, this meta-analysis may have been limited by the small number of eligible studies available. In particular, there were only four, three, and four publications available for rs3212961, rs3212948, rs2298882 polymorphisms, respectively, which may have attenuated the statistical power. Second, the missing data regarding cancer stage and insufficient histological details may also have impacts on the interpretation. Third, publication bias was detected by Egger's test, while analyzing the association between rs11615 polymorphism and lung cancer risk under the homozygous and dominant models. Forth, due to the deficiency of information on age, sex, ethnicity, and exposures (e.g., smoking, pack/year, and drinking), conclusions could only rely on unadjusted ORs, which might suffer confounding bias. Ideally, to perform a more precise analysis, crude ORs should be adjusted by potential confounders, such as age, sex, and other environmental factors after study results are pooled together. Unfortunately, in most cases, meta-analyses had to present only unadjusted ORs for the following reasons: 1) not all studies included in the meta-analysis provided adjusted ORs, 2) the ORs published in different studies were not adjusted by the same potential confounders, 3) usually only aggregate data (e.g., sex, age, and genotype) but not individual data are available in the reports. Given these deficits, we cannot eliminate the possibility that the significant associations observed in the current analysis might result from chance. Therefore, these results should be interpreted with caution. Further carefully designed studies with large sample sizes of different ethnic populations are warranted to validate our findings. Finally, this meta-analysis has been limited by the failure to acquire genotype data for some planned subgroup analyses. Particularly, *ERCC1* gene polymorphism may play roles to different extents in smoked-related and non-smoked-related lung cancer; however, very few of the studies in this meta-analysis separately reported SNP genotype counts for smokers and non-smokers. Future investigator should carefully list genotype counts for smokers and non-smokers to allow such stratified analyses to be done.

## Conclusion

Collectively, with more studies incorporated, we were able to conduct a more precise and robust evaluation for *ERCC1* rs11615 and rs3212986 polymorphisms. We found that there was null association between these two polymorphisms and lung cancer risk, and that *ERCC1* rs11615 may play a more profound role in lung cancer risk of non-smokers than that of smokers. Among the rest of polymorphisms, only rs3212948 polymorphism seemed to correlate with lung cancer susceptibility and act as a protective factor. Carefully designed studies with large sample size involving different ethnicity, smoking status, and cancer types are warranted to validate these findings.

## Supporting Information

Figure S1 ***ERCC1*** **mRNA expression by the genotypes of** ***ERCC1*** **rs3212948 polymorphism.**
(TIF)

Checklist S1 **PRISMA Checklist.**
(DOC)

## References

[1] Gorlova OY, Weng SF, Zhang Y, Amos CI, Spitz MR, et al (2008) DNA repair capacity and lung cancer risk in never smokers. Cancer Epidemiol Biomarkers Prev 17: 1322–1328. 18559546 10.1158/1055-9965.EPI-07-2591 PMC6587178

[2] Shen H, Spitz MR, Qiao Y, Guo Z, Wang LE, et al (2003) Smoking, DNA repair capacity and risk of nonsmall cell lung cancer. Int J Cancer 107: 84–88. 12925960 10.1002/ijc.11346

[3] Kietthubthew S, Sriplung H, Au WW, Ishida T (2006) Polymorphism in DNA repair genes and oral squamous cell carcinoma in Thailand. Int J Hyg Environ Health 209: 21–29. 16373199 10.1016/j.ijheh.2005.06.002

[4] Zienolddiny S, Campa D, Lind H, Ryberg D, Skaug V, et al (2006) Polymorphisms of DNA repair genes and risk of non-small cell lung cancer. Carcinogenesis 27: 560–567. 16195237 10.1093/carcin/bgi232

[5] Jones NR, Spratt TE, Berg AS, Muscat JE, Lazarus P, et al (2011) Association studies of excision repair cross-complementation group 1 (ERCC1) haplotypes with lung and head and neck cancer risk in a Caucasian population. Cancer Epidemiol 35: 175–181. 20863778 10.1016/j.canep.2010.08.007 PMC3081042

[6] Zhang L, Wang J, Xu L, Zhou J, Guan X, et al (2012) Nucleotide excision repair gene ERCC1 polymorphisms contribute to cancer susceptibility: a meta-analysis. Mutagenesis 27: 67–76. 22002622 10.1093/mutage/ger062

[7] Li Y, Gu S, Wu Q, Fu X, Mao Y, et al (2007) No association of ERCC1 C8092A and T19007C polymorphisms to cancer risk: a meta-analysis. Eur J Hum Genet 15: 967–973. 17522621 10.1038/sj.ejhg.5201855

[8] Cao C, Zhang YM, Wang R, Sun SF, Chen ZB, et al (2011) Excision repair cross complementation group 1 polymorphisms and lung cancer risk: a meta-analysis. Chin Med J (Engl) 124: 2203–2208. 21933627

[9] Holm K, Melum E, Franke A, Karlsen TH (2010) SNPexp - A web tool for calculating and visualizing correlation between HapMap genotypes and gene expression levels. BMC Bioinformatics 11: 600. 21167019 10.1186/1471-2105-11-600 PMC3022629

[10] Thorisson GA, Smith AV, Krishnan L, Stein LD (2005) The International HapMap Project Web site. Genome Res 15: 1592–1593. 16251469 10.1101/gr.4413105 PMC1310647

[11] Mantel N, Haenszel W (1959) Statistical aspects of the analysis of data from retrospective studies of disease. J Natl Cancer Inst 22: 719–748. 13655060

[12] DerSimonian R, Laird N (1986) Meta-analysis in clinical trials. Control Clin Trials 7: 177–188. 3802833 10.1016/0197-2456(86)90046-2

[13] Egger M, Davey Smith G, Schneider M, Minder C (1997) Bias in meta-analysis detected by a simple, graphical test. BMJ 315: 629–634. 9310563 10.1136/bmj.315.7109.629 PMC2127453

[14] He J, Shi TY, Zhu ML, Wang MY, Li QX, et al (2013) Associations of Lys939Gln and Ala499Val polymorphisms of the XPC gene with cancer susceptibility: a meta-analysis. Int J Cancer 133: 1765–1775. 23400628 10.1002/ijc.28089

[15] Zhou W, Liu G, Park S, Wang Z, Wain JC, et al (2005) Gene-smoking interaction associations for the ERCC1 polymorphisms in the risk of lung cancer. Cancer Epidemiol Biomarkers Prev 14: 491–496. 15734977 10.1158/1055-9965.EPI-04-0612

[16] Yu D, Zhang X, Liu J, Yuan P, Tan W, et al (2008) Characterization of functional excision repair cross-complementation group 1 variants and their association with lung cancer risk and prognosis. Clin Cancer Res 14: 2878–2886. 18451256 10.1158/1078-0432.CCR-07-1612

[17] Yin Z, Zhou B, He Q, Li M, Guan P, et al (2009) Association between polymorphisms in DNA repair genes and survival of non-smoking female patients with lung adenocarcinoma. BMC Cancer 9: 439. 20003463 10.1186/1471-2407-9-439 PMC2803496

[18] Yin J, Vogel U, Ma Y, Wang H, Wang C, et al (2012) A specific diplotype defined by PPP1R13L rs1970764, CD3EAP rs967591 and ERCC1 rs11615 and lung cancer risk in a Chinese population. Lung Cancer 76: 286–291. 22335888 10.1016/j.lungcan.2012.01.001

[19] Vogel U, Laros I, Jacobsen NR, Thomsen BL, Bak H, et al (2004) Two regions in chromosome 19q13.2-3 are associated with risk of lung cancer. Mutat Res 546: 65–74. 14757194 10.1016/j.mrfmmm.2003.11.001

[20] Sakoda LC, Loomis MM, Doherty JA, Julianto L, Barnett MJ, et al (2012) Germ line variation in nucleotide excision repair genes and lung cancer risk in smokers. Int J Mol Epidemiol Genet 3: 1–17. 22493747 PMC3316453

[21] Matullo G, Dunning AM, Guarrera S, Baynes C, Polidoro S, et al (2006) DNA repair polymorphisms and cancer risk in non-smokers in a cohort study. Carcinogenesis 27: 997–1007. 16308313 10.1093/carcin/bgi280

[22] Kazma R, Babron MC, Gaborieau V, Genin E, Brennan P, et al (2012) Lung cancer and DNA repair genes: multilevel association analysis from the International Lung Cancer Consortium. Carcinogenesis 33: 1059–1064. 22382497 10.1093/carcin/bgs116 PMC3334518

[23] Deng Q, Sheng L, Su D, Zhang L, Liu P, et al (2011) Genetic polymorphisms in ATM, ERCC1, APE1 and iASPP genes and lung cancer risk in a population of southeast China. Med Oncol 28: 667–672. 20354815 10.1007/s12032-010-9507-2

[24] Zhang Z, Zhou C, Zhang J, Tang L, Su B (2008) [Relationship between polymorphisms of DNA repair gene ERCC1 and susceptibility to lung cancer.]. Zhongguo Fei Ai Za Zhi 11: 183–188. 20731898 10.3779/j.issn.1009-3419.2008.02.022

[25] Yin J, Li J, Vogel U, Wang H (2005) Polymorphisms of DNA repair genes: ERCC1 G19007A and ERCC2/XPD C22541A in a northeastern Chinese population. Biochem Genet 43: 543–548. 16341770 10.1007/s10528-005-8170-3

[26] Yin J, Vogel U, Guo L, Ma Y, Wang H (2006) Lack of association between DNA repair gene ERCC1 polymorphism and risk of lung cancer in a Chinese population. Cancer Genet Cytogenet 164: 66–70. 16364765 10.1016/j.cancergencyto.2005.07.003

[27] Yin J, Vogel U, Ma Y, Qi R, Wang H (2008) Haplotypes of nine single nucleotide polymorphisms on chromosome 19q13.2-3 associated with susceptibility of lung cancer in a Chinese population. Mutat Res 641: 12–18. 18358500 10.1016/j.mrfmmm.2008.02.004

[28] Yin J, Vogel U, Ma Y, Qi R, Wang H, et al (2011) HapMap-based study of a region encompassing ERCC1 and ERCC2 related to lung cancer susceptibility in a Chinese population. Mutat Res 713: 1–7. 21601580 10.1016/j.mrfmmm.2011.05.003

[29] Ma H, Xu L, Yuan J, Shao M, Hu Z, et al (2007) Tagging single nucleotide polymorphisms in excision repair cross-complementing group 1 (ERCC1) and risk of primary lung cancer in a Chinese population. Pharmacogenet Genomics 17: 417–423. 17502833 10.1097/01.fpc.0000239975.77088.17

[30] Shen M, Berndt SI, Rothman N, Demarini DM, Mumford JL, et al (2005) Polymorphisms in the DNA nucleotide excision repair genes and lung cancer risk in Xuan Wei, China. Int J Cancer 116: 768–773. 15849729 10.1002/ijc.21117

[31] Kang H, Zhao JH, Zhang JJ (2011) Single nucleotide polymorphysims of CYP2D6 gene G4268C and ERCC1 gone C8092A and their genetic susceptibility to lung cancer. Modern Oncology 19: 1275–1279.

[32] He J, Qiu LX, Wang MY, Hua RX, Zhang RX, et al (2012) Polymorphisms in the XPG gene and risk of gastric cancer in Chinese populations. Hum Genet 131: 1235–1244. 22371296 10.1007/s00439-012-1152-8

