# Supplementary figures and images for: Association Studies of ERCC1 Polymorphisms with Lung Cancer Susceptibility: A Systematic Review and Meta-Analysis
Source: PLoS One. 2014 May 19;9(5):e97616. doi: 10.1371/journal.pone.0097616 (PMC4026486; doi:10.1371/journal.pone.0097616)

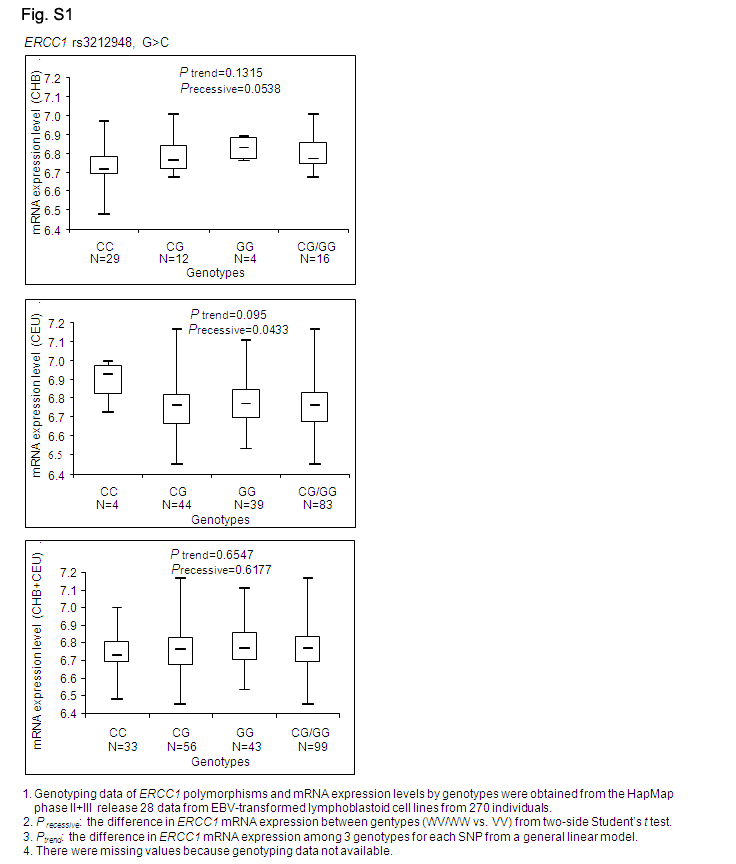

Supplement: Figure S1 — ERCC1 mRNA expression by the genotypes of ERCC1 rs3212948 polymorphism. (TIF) [file pone.0097616.s001.tif]
